# FANCD2-Associated Nuclease 1 Partially Compensates for the Lack of Exonuclease 1 in Mismatch Repair

**DOI:** 10.1128/MCB.00303-21

**Published:** 2021-08-24

**Authors:** Katja Kratz, Mariela Artola-Borán, Saho Kobayashi-Era, Gene Koh, Goncalo Oliveira, Shunsuke Kobayashi, Andreia Oliveira, Xueqing Zou, Julia Richter, Masataka Tsuda, Hiroyuki Sasanuma, Shunichi Takeda, Joanna I. Loizou, Alessandro A. Sartori, Serena Nik-Zainal, Josef Jiricny

**Affiliations:** a Institute of Molecular Cancer Research, University of Zurich, Zurich, Switzerland; b Institute of Biochemistry of the ETH Zurich, Zurich, Switzerland; c Wellcome Sanger Institute, Wellcome Genome Campus, Hinxton, United Kingdom; d Academic Department of Medical Genetics, The Clinical School, University of Cambridge, Cambridge, United Kingdom; e MRC Cancer Unit, The Clinical School, University of Cambridge, Cambridge, United Kingdom; f Institute of Cancer Research, Department of Medicine I, Comprehensive Cancer Centre, Medical University of Vienna, Vienna, Austria; g Department of Radiation Genetics, Graduate School of Medicine, Kyoto University, Kyoto, Japan

**Keywords:** DNA repair, EXO1, exonuclease, FAN1, mismatch repair, MLH1, MSH6, mutational signature

## Abstract

Germline mutations in the mismatch repair (MMR) genes *MSH2*, *MSH6*, *MLH1*, and *PMS2* are linked to cancer of the colon and other organs, characterized by microsatellite instability and a large increase in mutation frequency. Unexpectedly, mutations in *EXO1*, encoding the only exonuclease genetically implicated in MMR, are not linked to familial cancer and cause a substantially weaker mutator phenotype. This difference could be explained if eukaryotic cells possessed additional exonucleases redundant with EXO1. Analysis of the MLH1 interactome identified FANCD2-associated nuclease 1 (FAN1), a novel enzyme with biochemical properties resembling EXO1. We now show that FAN1 efficiently substitutes for EXO1 in MMR assays and that this functional complementation is modulated by its interaction with MLH1. FAN1 also contributes to MMR *in vivo*; cells lacking both EXO1 and FAN1 have an MMR defect and display resistance to *N*-methyl-*N*-nitrosourea (MNU) and 6-thioguanine (TG). Moreover, FAN1 loss amplifies the mutational profile of EXO1-deficient cells, suggesting that the two nucleases act redundantly in the same antimutagenic pathway. However, the increased drug resistance and mutator phenotype of FAN1/EXO1-deficient cells are less prominent than those seen in cells lacking MSH6 or MLH1. Eukaryotic cells thus apparently possess additional mechanisms that compensate for the loss of EXO1.

## INTRODUCTION

Lynch syndrome, also known as hereditary nonpolyposis colon cancer (HNPCC), is an inherited predisposition to cancer of the colon, endometrium, ovary, and other organs that is linked to mutations in mismatch repair (MMR) genes (1). Loss of MMR brings about an increase in spontaneous mutation rates, as well as microsatellite instability (MSI) (2, 3). Importantly, the magnitude of the mutator phenotype is dependent on the *MMR* gene that is mutated. Thus, compared to MMR-proficient cells, up to 100-fold higher rates of substitution mutations and high MSI are associated with loss of *MSH2*, *MLH1*, or *PMS2*, whereas inactivation of *MSH6* causes a similar increase in substitution mutations, but MSI is limited largely to mononucleotide repeats. This is due to the partial functional redundancy between MSH6 and MSH3 in the mismatch recognition factors MutSα (MSH2/MSH6) (4–6) and MutSβ (MSH2/MSH3) (7). Unexpectedly, loss-of-function mutations in *EXO1*, a gene encoding the only exonuclease genetically implicated in eukaryotic MMR to date (8), was shown to cause only a weak mutator phenotype in Saccharomyces cerevisiae (9) and mice (10). Although *EXO1^−/−^* mice were reported to acquire lymphomas late in life (10, 11), the gene does not appear to be mutated in HNPCC patients (12). It had therefore been postulated that MMR in eukaryotes is likely to involve another, as yet unidentified, nuclease that might compensate for the lack of EXO1 (see references 13 and 14 for reviews).

EXO1 acts downstream from mismatch recognition, mediated primarily by MutSα. Mismatch-bound MutSα undergoes an ATP-dependent conformational change that enables it to recruit MutLα and interact with PCNA/RFC bound at a strand discontinuity (e.g., a terminus of an Okazaki fragment or a nick introduced by activated MutLα). This leads to the recruitment and stimulation of EXO1, which subsequently degrades the discontinuous strand in a 5′ to 3′ direction. Once the mispair is removed, EXO1 is inhibited (15, 16), the single-stranded gap is filled in by polymerase δ, and the remaining nick is sealed by DNA ligase I (reviewed in references 17 and 18).

**FIG 1:**
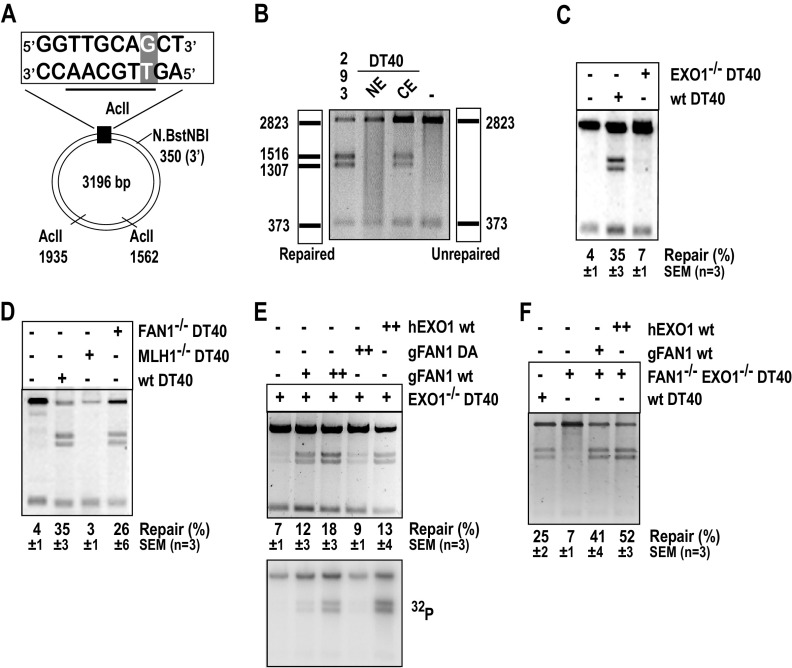
Recombinant FAN1 or EXO1 rescue the MMR defect in extracts of *EXO1^−/−^* or *EXO1^−/−^ FAN1^−/−^* chicken B-lymphocyte DT40 cells. (A) Scheme of the substrate used in the *in vitro* MMR assays. The T/G mismatch at position 46 of the phagemid heteroduplex makes it refractory to AclII cleavage at this site. Digestion with AclII generates two fragments, of 2,823 and 373 bp (panel B, right scheme). Repair of the Nt.BstNBI-nicked T/G substrate to T/A regenerates the AclII cleavage site. AclII digest thus yields two additional bands, of 1,516 and 1,307 bp (panel B, left scheme). Repair efficiency was defined as the ratio of the intensities of the 1,516-bp versus the 2,823-bp band in GelRed-stained agarose gels. (B) MMR assay with DT40 nuclear (NE) and cytoplasmic (CE) extracts. MMR-proficient HEK293T-MutLα(+) nuclear extract was used as the positive control (293). Incubation of the substrate in MMR buffer without extract was used as the negative control (–). (C) *EXO1^−/−^* DT40 cell extracts are largely MMR-deficient. (D) *FAN1^−/−^* DT40 extracts are MMR-proficient, unlike extracts of MLH1^−/−^ DT40 cells. (E) MMR in EXO1-deficient DT40 extracts can be rescued by recombinant human EXO1 (hEXO1, 400 ng [++]) and chicken FAN1 (gFAN1, 80 ng [+] or 160 ng [++]) but not the nuclease-dead gFAN1 D977A (160 ng [++]) variant. The bottom panel is an autoradiograph of the same gel. (F) Recombinant hEXO1 (400 ng [++]) and gFAN1 (80 ng [+]) can rescue MMR in *FAN1^−/−^ EXO1^−/−^* DT40 extracts. Panels B to F show images of representative GelRed-stained 1% agarose gels run at 200 V in TAE buffer.

The above-described mechanism has been deduced from a large number of biochemical investigations that made use of a nicked mismatch-containing plasmid substrate (similar to that shown in Fig. 1A) and extracts of MMR-proficient or -deficient cells (19, 20). These efforts culminated in the reconstitution of the minimal human MMR system consisting of MutSα, MutLα, RFC, PCNA, replication protein A (RPA), DNA polymerase δ, EXO1, ATP, and deoxynucleoside triphosphate (dNTPs) (21, 22).

In an attempt to learn more about the composition of the mismatch repairosome, we carried out proteomic analyses of MMR complexes isolated from cells by affinity chromatography. Using MLH1 as bait, we identified *KIAA1018* (23), which encodes a 5′ flap endonuclease and a 5′ to 3′ exonuclease. Given the similarity of its biochemical properties to EXO1, we inactivated the *KIAA1018* gene in chicken DT40 cells and tested the sensitivity of the knockout cells to a variety of DNA-damaging agents. Unexpectedly, the cells were hypersensitive to the DNA interstrand cross-linking (ICL) agents cisplatin and mitomycin C (MMC) (24), a characteristic trait of cells of Fanconi anemia patients (25). This finding, further substantiated and extended in human cells (26–29), led to changing the name of the KIAA1018 protein to FAN1 (FANCD2-associated nuclease 1). But if FAN1 participates in ICL repair, why does it need to interact with MLH1? Our preliminary studies revealed that MutLα was necessary neither to recruit FAN1 to ICLs nor to modulate its nuclease activity (26). Could the interaction be required for FAN1 to participate in MMR, possibly as a backup for EXO1?

In an attempt to answer this question, we first set out to examine the MMR proficiency of extracts of chicken B-lymphocyte DT40 and human cells lacking EXO1, FAN1, or both proteins. Here, we show that FAN1 can rescue the MMR defect in EXO1-deficient extracts and that the efficiency of the rescue is augmented by its interaction with MLH1. *In vivo*, MMR efficiency of cells lacking both EXO1 and FAN1 was lower than that of the single mutants, and the double mutant cells were more resistant to *N*-methyl-*N*-nitrosourea (MNU) or 6-thioguanine (TG) than cells lacking only one of these enzymes (resistance to these agents is a hallmark of MMR-deficient cells). We further show that the loss of FAN1 amplifies the mutational signature of *EXO1* knockout cells, which implicates the two polypeptides in the same antimutagenic pathway.

## RESULTS

Having discovered FAN1/KIAA1018 as an interactor of MLH1 (23) and having shown that it is a 5′-flap endonuclease and a 5′-3′ exonuclease (26, 30), we hypothesized that the protein might play a role in MMR as a backup for EXO1, which has similar activities (reviewed in references 13 and 14) and which also interacts with MLH1 (31–33). To test this hypothesis, we first set out to assemble a set of genetically homogeneous cell lines disrupted at the *MLH1*, *EXO1*, and *FAN1* loci. We also wished to test the phenotype of the *FAN/EXO1* double mutant. Because some of these cell lines had in the past been generated in our laboratories in chicken DT40 and human TSCER2 (here referred to as TK6) backgrounds, we decided to complete the set in these two B cell types.

### Generation of *MLH1^−/−/−^* and *FAN1^−/−^ EXO1^−/−^* DT40 cell lines.

To disrupt the chicken *MLH1* gene (see Fig. S1A to C in the supplemental material), we used a protocol employed in the generation of the *EXO1* (34) and *FAN1* (24) loci in DT40 cells. We introduced stop codons and antibiotic selection markers into exon 6, which contains the MLH1 ATP binding site. Because the *MLH1* locus lies on chromosome 2, which is present in three copies in DT40 cells, we generated gene-targeting constructs carrying three different selection markers (Fig. S1A). We isolated clones lacking one, two, and three alleles, as shown by Southern blotting (Fig. S1B) and reverse transcriptase PCR (RT-PCR) (Fig. S1C) analyses. We also disrupted the *EXO1* gene in *FAN1^−/−^* cells (24) by replacing exons 3 to 7 (34) with antibiotic selection markers (Fig. S1D) to generate the *FAN1^−/−^ EXO1^−/−^* double knockout cells. As described above, knockout of the wild-type (WT)-*EXO1* alleles was confirmed by Southern blotting (Fig. S1E) and RT-PCR (Fig. S1F) analyses.

### *MLH1^−/−/−^* and *EXO1^−/−^* DT40 extracts are MMR-deficient.

We first set out to study the relative contributions of the two nucleases to MMR efficiency using *in vitro* complementation assays (35) and the heteroduplex substrate shown in Fig. 1A. Unexpectedly, nuclear extracts of WT DT40 cells prepared following our standard protocol (36) contained high protein concentrations (Fig. S1G, lane NEa) but also fragmented, possibly apoptotic genomic DNA (Fig. S1H, lane NEa). We therefore modified the protocol (see Materials and Methods) and obtained nuclear and cytoplasmic extracts that contained lower protein levels (Fig. S1G, lanes NE and CE, respectively) but substantially less genomic DNA (Fig. S1H, lanes NE and CE). No repair of the G/T mismatch was detectable when the nuclear extracts (Fig. 1B, lane NE) were used in the *in vitro* MMR assay (36). In contrast, the cytoplasmic fraction was MMR-proficient (Fig. 1B, lane CE), as reported also for cytoplasmic extracts of human cells (37). Although the repair efficiency was lower than in nuclear extracts of HEK293Lα cells (Fig. 1B, lane 293), we used the cytoplasmic DT40 extracts in subsequent experiments.

Similar to extracts of *Exo1^−/−^* mouse embryonic fibroblasts (MEFs) (10, 11, 38) and human HEK293 cells depleted of EXO1 with small interfering RNA (siRNA) (35), MMR activity of the DT40 *EXO1^−/−^* extracts was only slightly higher than that in extracts lacking MLH1 (Fig. 1, compare panels C and D, respectively). In contrast, cytoplasmic extracts of *FAN1^−/−^* DT40 cells (24) were MMR-proficient (Fig. 1D). This confirms that EXO1 is the major MMR-associated nuclease in this *in vitro* assay.

### Recombinant FAN1 rescues MMR in EXO1-deficient DT40 cell extracts.

The above-described results indicated that although FAN1 was present in the EXO1-deficient extracts, it did not detectably contribute to MMR in this *in vitro* assay. However, we considered the possibility that the endogenous protein in the extracts was inactivated (e.g., by posttranslational modifications) or sequestered in an inactive complex. In order to learn whether exogenous FAN1 could compensate for EXO1 in this assay, we supplemented the EXO1-deficient extract with purified recombinant chicken FAN1. As shown in Fig. 1E (top panel), WT gFAN1, but not its nuclease-dead D977A (DA) variant, efficiently rescued the MMR defect of EXO1-deficient cell extracts. In order to ascertain that the repair of the G/T mismatch in the substrate occurred by the canonical MMR pathway, which involves exonucleolytic degradation of the nicked strand followed by repair synthesis, we carried out the assay in the presence of [α-^32^P]dATP. As shown in the bottom panel of Fig. 1E, similar amounts of the radionuclide were incorporated into the 1,516- and 1,307-bp fragments in extracts complemented with the WT FAN1, but not with the nuclease-dead FAN1 variant. This is indicative of canonical MMR. The extract was complemented with excess recombinant purified human EXO1, despite the low degree of homology (57.6% identity) between the chicken and human polypeptides (Fig. 1E). Similarly, extracts of DT40 cells lacking both EXO1 and FAN1 were also complemented with both nucleases (Fig. 1F).

### FAN1 complements the MMR defect in EXO1-deficient extracts of human cells, and this function is augmented by its interaction with MLH1.

**FIG 2:**
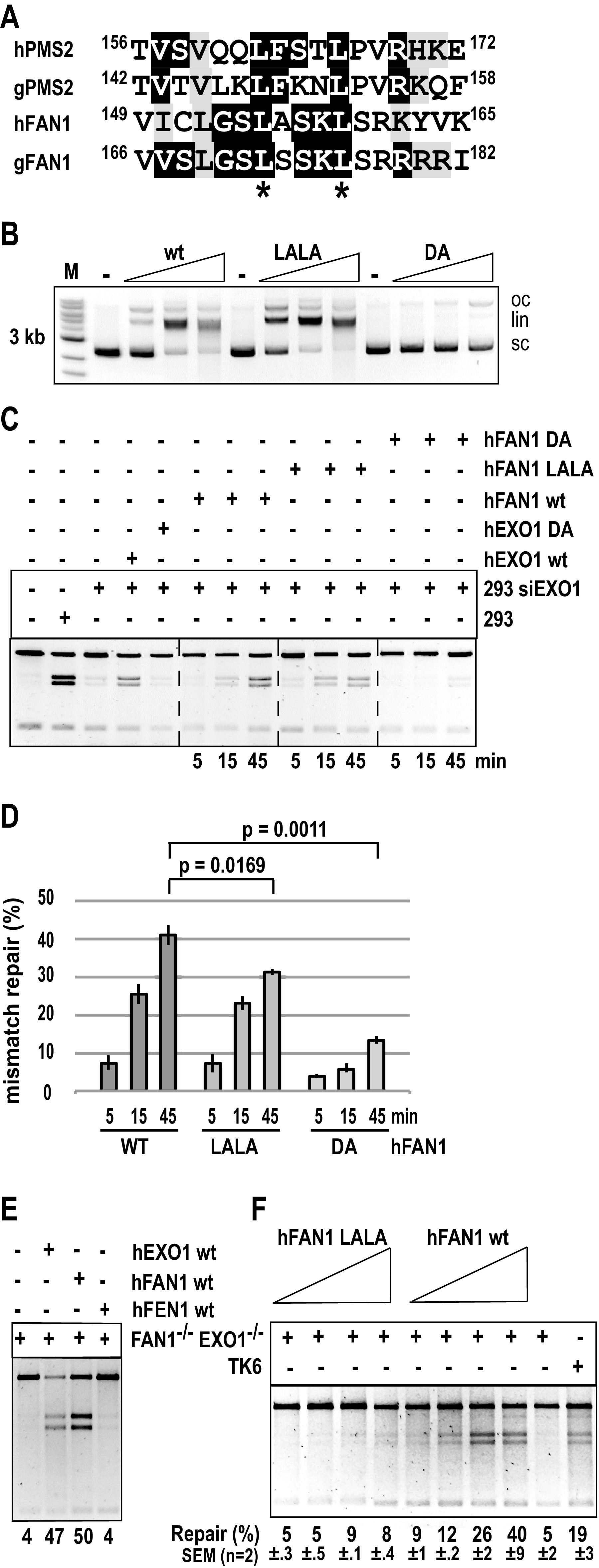
The ability of FAN1 to rescue the MMR defect in human cells lacking EXO1 or both EXO1 and FAN1 is affected by FAN1 interaction with MLH1. (A) Alignment of the putative MLH1 interaction motifs in human (h) and chicken (g) FAN1 and PMS2. The conserved leucines that were mutated to alanines are marked with asterisks. (B) WT FAN1 and its LALA variant have comparable activities in a nonspecific nuclease assay, which measures the relaxation and cleavage of a supercoiled plasmid substrate. The FAN1 DA nuclease-dead mutant was included as the negative control. Oc, open circular; lin, linear; sc, supercoiled plasmid. (C) WT EXO1 or FAN1, but not the respective nuclease-dead DA mutants, can rescue the MMR activity of HEK293 extracts in which EXO1 was depleted with siRNA (Fig. S2A). The activity of the LALA variant in this MMR assay was diminished compared to the WT protein. (D) Quantitation of the time course shown in panel C. (E) Recombinant human FAN1 or EXO1, but not FEN1, rescues the MMR defect in nuclear extracts of *FAN1^−/−^ EXO1^−/−^* TK6 cells. (F) Comparison of the ability of equal amounts of WT FAN1 and its LALA variant to complement the *in vitro* MMR deficiency of FAN1/EXO1-deficient TK6 cells. Panels B, C, E, and F show representative images of GelRed-stained 1% agarose gels run at 200V in TAE buffer.

To confirm that the above-described findings were not confined to chicken DT40 cells, we set out to repeat the *in vitro* MMR experiments using extracts of human cells and recombinant human proteins. In addition, given that FAN1 was identified as an MLH1-interacting protein (23), we wanted to test whether its ability to complement the MMR defect in EXO1-deficient cell extracts was dependent on this interaction. An *In silico* search for a possible MLH1 interaction motif identified a short amino acid sequence that is conserved in the human (h) and chicken (g) FAN1 proteins, as well as in PMS2, the heterodimeric partner of MLH1 in MutLα (Fig. 2A). In order to test whether this motif was necessary for the interaction between FAN1 and MLH1, we expressed in HEK293 cells green fluorescent protein (GFP)-tagged FAN1, as well as its LALA variant that carried L155A and L159A mutations in this motif. As shown in Fig. S2A, eluent from incubation of GFP-FAN1 cell extracts with GFP-TRAP beads contained MLH1, but this was not the case when extracts of cells expressing the GFP-FAN1-LALA mutant were used. This implicated the two above-described leucines in interaction with MLH1.

We then expressed and purified (26) the human FAN1 WT, LALA, and nuclease-dead D960A variants in the baculovirus system (Fig. S2B) and compared their activities in a nonspecific endonuclease assay, in which we monitored the relaxation and cleavage of a supercoiled plasmid substrate (39). The L155A/L159A mutations did not affect the nucleolytic activity of FAN1, whereas the D960A variant was largely inactive (Fig. 2B). We therefore decided to compare the ability of equal concentrations of the three variants to rescue the MMR defect in EXO1-depleted extracts of HEK293 cells. As shown in Fig. S2C, EXO1 was not detected in the siRNA-treated HEK293 extracts by Western blotting, while FAN1 levels remained unaltered by the siRNA treatment. As anticipated, MMR efficiency in the depleted extracts was substantially diminished, and the defect was largely restored by the addition of purified recombinant WT EXO1 but not the nuclease-dead D173A mutant (35). The extracts were complemented also by WT FAN1 but not by the nuclease-dead D960A (Fig. 2C). A time course experiment showed that the FAN1 LALA mutant was less efficient in complementing the MMR defect in the EXO1-depleted extracts than the WT protein (Fig. 2C and D).

To generate *FAN1* and *EXO1* knockouts in the TK6 B cell line, we used CRISPR/Cas9 or transcription activator-like effector nucleases (TALEN) to generate locus-specific double-strand breaks, which substantially increased the efficiency of gene disruption. Using the constructs shown in Fig. S2D and F, we generated knockout clones lacking FAN1 and EXO1, respectively. The disruptions were verified by Southern blotting (Fig. S2E and G) and Western blotting (Fig. S2H), as well as by genomic sequencing. We also knocked out EXO1 in the FAN1^−/−^ TK6 cells (Fig. S2H). MLH1-deficient cells were generated previously (40).

As shown in Fig. 2E, TK6 extracts lacking both FAN1 and EXO1 were MMR-deficient but could be complemented with either recombinant enzyme. It could be argued that the ability of FAN1 to complement the MMR defect in EXO1-deficient extracts might be nonspecific, in other words, that the EXO1 defect might be complemented by any 5′ to 3′ nuclease capable of degrading DNA from a single-stranded nick. We are not aware of human nucleases possessing such activity, other than EXO1 and FAN1, but we cannot exclude the possibility that they exist. However, we wanted to test whether the EXO1/FAN1-deficient extracts could be complemented by FEN1, a 5′-flap endonuclease that participates in the EXO1-independent, strand displacement-mediated MMR described previously (38). This was not the case (Fig. 2E).

We also tested the ability of the FAN1 LALA variant to complement the double-deficient extracts. As in the case of HEK293 EXO1-depleted extracts, the LALA variant was less efficient in complementing the MMR defect than the WT protein (Fig. 2F). Taken together, the above-described biochemical evidence showed that FAN1 can substitute for EXO1 *in vitro* and that the FAN1-MLH1 interaction augments FAN1 activity in MMR.

### FAN1 contributes to repair efficiency also in an *in vivo* MMR assay.

**FIG 3:**
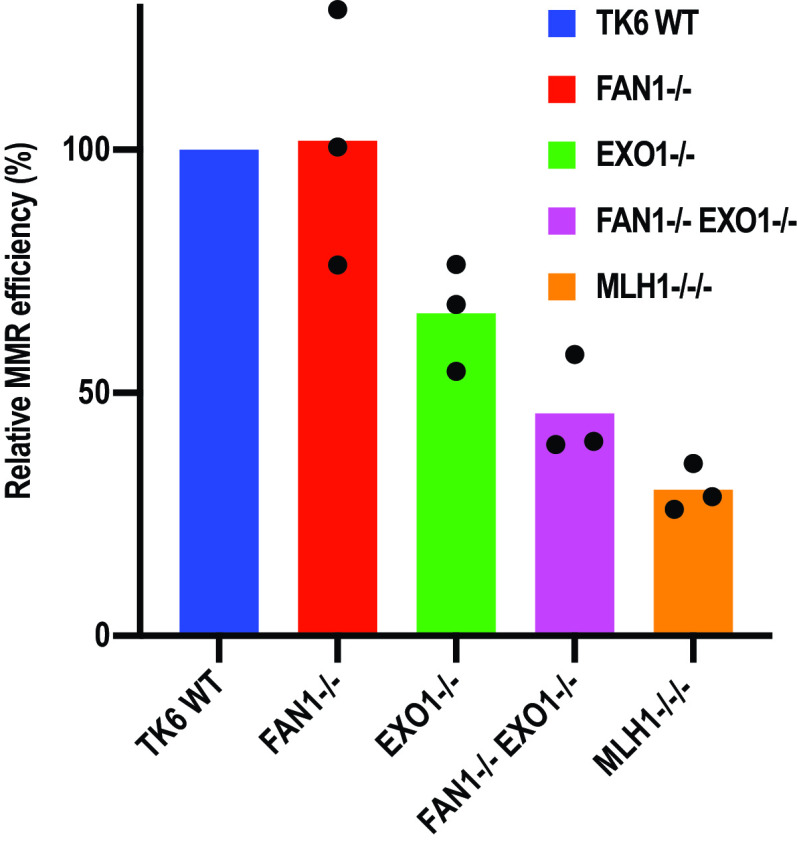
FAN1 contributes to MMR efficiency *in vivo*. The indicated TK6 cell lines were cotransfected with an EGFP control plasmid and an equal amount of a reporter plasmid containing a T/G mismatch in the mCherry gene, as well as a nick in the T-strand. MMR efficiency was estimated from the ratio of the EGFP (green) and the mCherry (red) signal generated by correction of the T/G mismatch to C/G, which converts a TAG stop codon in the mCherry ORF to a TGG Trp codon. The MMR efficiency of the mutants is shown relative to WT cells, which were arbitrarily set to 100%. The results of three independent experiments are shown.

To test whether FAN1 can compensate for the lack of EXO1 also *in vivo*, we deployed an MMR assay that makes use of fluorescent reporter plasmids similar to those used in the *in vitro* assays. This experiment is based on the cotransfection into the different cell lines of an enhanced GFP (EGFP) control vector, together with a heteroduplex containing a T/G mismatch and a nick in the T strand in the open reading frame (ORF) of the pmCherry reporter gene (see Materials and Methods). Correction of the T/G mismatch to C/G and thus of a TAG (stop) to a TGG (Trp) codon restores the pmCherry expression (41). After cotransfection of an equimolar mixture of the two plasmids, MMR efficiency was measured by a fluorescence-activated cell sorter (FACS) as the ratio of red (pmCherry) versus green (EGFP) fluorescence. In control experiments, about 80% of EGFP-positive cells transfected with the positive control, the C/G pmCherry plasmid, were red fluorescence positive, and no red signal was seen following transfection with the negative control, the T/A pmCherry vector. With conversion efficiency of the T/G mismatch to C/G in WT TK6 cells set to 100%, the relative efficiency in the FAN1-deficient cells was similar to that of WT cells. *EXO1^−/−^* cells displayed a slight MMR defect, with ∼70% of pmCherry-positive cells. MMR efficiency in the *FAN1*/*EXO1* double knockout cells was further decreased to ∼45%, and in MLH1-deficient cells, the red signal decreased further, to ∼20% of that seen in WT cells (Fig. 3). Taken together, the results of the above-described assays indicate that the lack of FAN1 does not affect MMR efficiency in the presence of EXO1 but that it can—at least partially—compensate for the lack of EXO1, both *in vivo* and *in vitro*. As both the above-described assays measure MMR efficiency on an artificial heteroduplex substrate, we set out to obtain additional *in vivo* evidence of the EXO1/FAN1 redundancy during MMR in genomic DNA.

### FAN1 deficiency augments the resistance of *EXO1^−/−^* cells to MNU and TG.

The MMR system has been shown to address also deoxyguanosines and thiodeoxyguanosines methylated at the *O*^6^ position of guanine by S_n_1-type methylating agents such as MNU, *N*-methyl-*N*-nitro-*N′*-nitrosoguanidine (MNNG), or temozolomide. *O^6^*-Methyl-2′-deoxyguanosine (^me^G) (42–44) and *S^6^*-methyl-2*′*-deoxy-6-thioguanosine (^me^TG) (45) can base pair with both T and C to form mismatch-like structures that trigger MMR. However, because excision is directed to the nascent strand, the modified nucleotides persist in the template. As a consequence, filling in of the repair patch generated by MMR excision regenerates the methylated mispairs, which results in futile repair synthesis, double-strand break (DSB) generation, and cytotoxicity (43, 46, 47). Because MMR-deficient cells do not process ^me^G- and ^me^TG-containing mispairs, they are generally more resistant than MMR-proficient cells to killing by S_n_1-type methylating agents and TG (48, 49).

**FIG 4:**
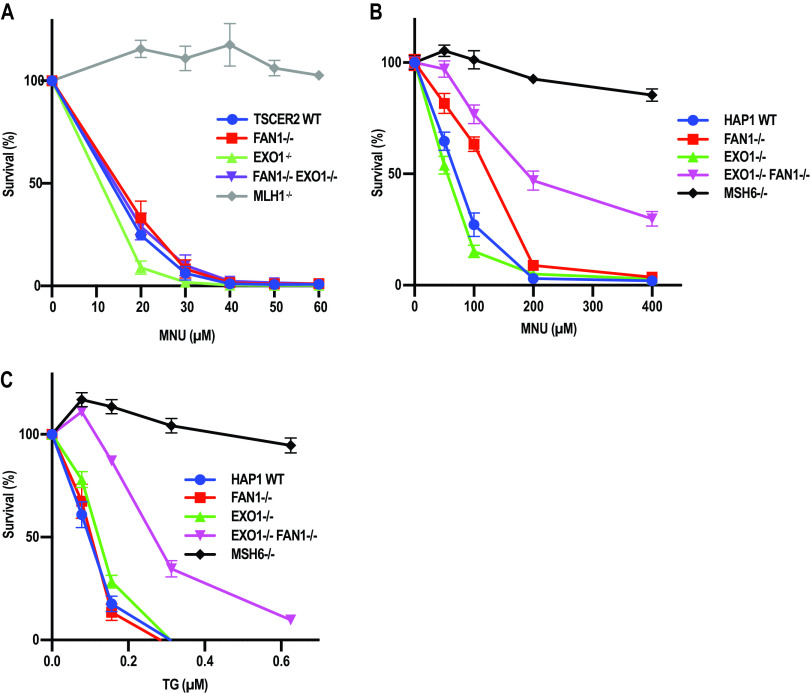
FAN1 deficiency augments the resistance of EXO1-deficient TK6 and HAP1 cells to MNU and TG. (A) Clonogenic assay showing the response of WT, *FAN1^−/−^*, *EXO1^−/−^*, and *FAN1^−/−^ EXO1^−/−^* TK6 cells to MNU. (B and C) MTT assays showing the response of WT, *FAN1^−^*, *EXO1^−^*, *EXO1^−^ FAN1^−^* and *MSH6^−^* HAP1 cells to MNU (B) and TG (C). The results of three independent experiments each carried out in triplicate are shown. Error bars represent the standard error of the mean.

To test the involvement of FAN1 in MMR-mediated processing of methylation damage, we treated TK6 WT, *EXO1^−/−^*, *FAN1^−/−^*, and *FAN1^−/−^ EXO1^−/−^* cells with MNU (Fig. 4A). As anticipated, MLH1-deficient cells were resistant to the chemical, unlike the WT or the FAN1-deficient cells. In contrast to *Mgmt^−/−^ Exo1^−/−^* mouse embryonic fibroblasts (50), which were reported to be slightly resistant to MNU, TK6 *EXO1^−/−^* cells were more sensitive to it than WT cells. Given the facts that B cells generally display high levels of recombination, that futile MMR leads to the generation of DSBs (43, 46, 47), and that EXO1 is involved in the long-range resection of DSBs (14, 51), this result was not unexpected. Importantly, the hypersensitivity of the *EXO1^−/−^* cells was rescued by exogenous expression of EXO1 (Fig. S3A and B), so the sensitive phenotype was not caused by some unscheduled mutation arising during the generation of these knockout cells.

Disruption of the *FAN1* gene in the *EXO1^−/−^* TK6 cells rescued their sensitivity (Fig. 4A), which suggests that FAN1 contributes to the cytotoxicity caused by methylating agents such as MNU and that its absence leads to a reduction in the generation of toxic DSBs. In support of this hypothesis, we observed that FAN1 can cleave DNA opposite a nick to generate a DSB (Fig. S3C). It is thus likely that FAN1 contributes to the MNU sensitivity of cells through cleaving DNA at the single-stranded gaps generated during futile MMR (46), and its absence results in slight resistance to this agent. Moreover, the rescue effect was specific to MNU; as shown in Fig. S3D, the *FAN1^−/−^ EXO1^−/−^* cells were more sensitive than either *EXO1^−/−^* or *FAN1^−/−^* cells to the interstrand cross-linking agent mitomycin C (MMC).

In order to confirm that the hypersensitivity of the *EXO1^−/−^* TK6 cells to MNU was cell type specific, we generated *FAN1^−^* and *EXO1^−^*/*FAN1^−^* lines in the haploid human HAP1 cell line and tested their sensitivity to this chemical, together with cells lacking EXO1 or MSH6 (52). Compared to TK6, the HAP1 cells were generally more resistant to MNU, possibly due to high levels of MGMT that were not neutralized by the *O^6^*-benzylguanine pretreatment (compare Fig. 4A and B). However, as anticipated, the MMR-deficient HAP1 cells were substantially more resistant to MNU than the wild-type controls. Like TK6, the HAP1 *EXO1^−^* cells were more sensitive than the wild-type cells, but this difference was not significant. Interestingly, the *FAN1^−^* cells displayed slight but reproducible resistance to MNU. This resistance was much more pronounced in the *EXO1^−^*/*FAN1^−^* cells, although it did not reach the extent seen in the MMR-deficient control cells (Fig. 4B).

The observed resistance of HAP1 *FAN1^−^* cells to MNU was unexpected. We wanted to explore the possibility that it was been linked to the high concentration of MNU used, which would have caused a substantial number of DNA breaks at abasic sites resulting from the removal of *N^3^*-methyladenine and *N^7^*-methylguanine by methyladenine glycosylase (53). As shown in Fig. S3C, FAN1 can cleave double-stranded DNA opposite a strand discontinuity, which would give rise to cytotoxicity. In order to substantiate this hypothesis, we treated the HAP1 cells with TG, which does not cause any damage to DNA bases and the toxicity of which is linked largely to MMR processing. As in the case of TK6 cells, the HAP1 *FAN1^−^* cells were similarly sensitive to the wild-type control and *EXO1^−^* cells, whereas the *EXO1^−^*/*FAN1^−^* cells were relatively resistant to TG (Fig. 4C). Together, the above results further confirm that FAN1 can compensate in MMR for the loss of EXO1 not only *in vitro* but also *in vivo*.

### FAN1 defect amplifies the mutational profile of EXO1-deficient cells.

**FIG 5:**
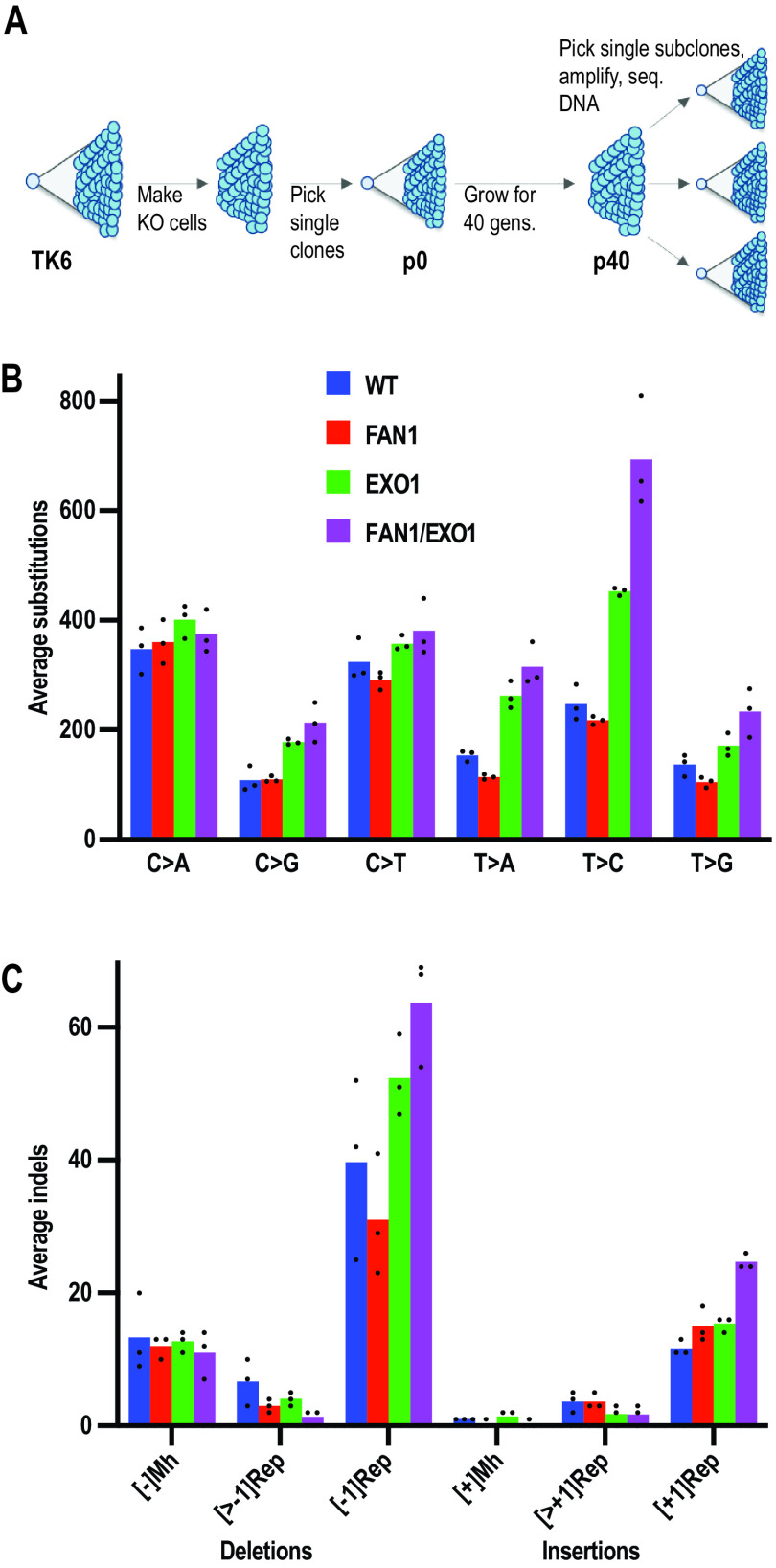
Mutational signatures of WT, *FAN1^−/−^*, *EXO1^−/−^*, and *FAN1^−/−^ EXO1^−/−^* TK6 cells. (A) Scheme of the experiment. After generation of the knockout cell lines, single cell clones of each genotype were isolated and expanded. A portion of these cells was used to isolate DNA for sequencing (p0), and the remainder was grown for 40 cell divisions (p40) to permit mutation accumulation. Three single clones from each of the p40 populations were then picked and expanded, and their DNAs were subjected to whole-genome sequencing. (B) Summary of substitution mutations in the indicated cell lines. (C) Summary of insertion and deletion mutations in the indicated cell lines. [±1Rep], insertion/deletion of a single nucleotide in a mononucleotide repeat; [>±1Rep], insertion/deletion of more than one nucleotide in a mononucleotide repeat; Mh, microhomology-directed insertion/deletion. The columns represent an average mutation count in three independent clones.

We wanted to obtain evidence of FAN1 involvement in spontaneous mutagenesis, a process in which MMR plays a key preventive role. Recent advances in genomic sequencing technology allow for the analysis of genomic changes, the so-called mutational signatures, at nucleotide resolution (52, 54). We therefore set out to determine the mutational profiles of our WT, *EXO1^−/−^*, *FAN1^−/−^*, and *FAN1^−/−^ EXO1^−/−^* TK6 cells. We measured the doubling times of the latter cell lines, isolated single cell clones of each genotype (p0), and grew them for exactly 40 cell generations to permit mutation accumulation. We then picked three single clones from each p40 population and expanded them just enough to harvest an adequate amount of DNA for whole-genome sequencing (Fig. 5A). Unique somatic mutations that had accumulated over the mutation accumulation period were detected by subtracting the mutations present at p0 from each of the p40 daughter subclones.

As shown in Fig. 5B, the substitution mutation profiles of the WT and *FAN1^−/−^* cells were largely similar for all six types of transitions and transversions. DNA of EXO1-deficient cells contained more substitutions of all types, the most prominent increase being that of T > C (A > G) transitions. This mutation is indicative of a defect in the correction of T/G mispairs, which arise most frequently as errors of DNA polymerases. The latter increase was further amplified in the DNA of FAN1 EXO1 double mutant cells, which contained slightly more substitutions of all other types, with the possible exception of C > A (G > T) transversions. The latter mutations are a characteristic footprint of oxidative DNA damage, caused by the mispairing of 8-oxoguanine with adenine during replication. This finding was not unexpected, given that 8-oxoG/A mispairs are generally addressed by the OGG1/MYH-dependent base excision repair rather than by MMR (55).

The loss of FAN1 also increased the frequency of single nucleotide deletions in mononucleotide repeats in EXO1-deficient cells (Fig. 5C). This finding strengthens the evidence of FAN1 involvement in MMR, given that deletions of A_*n*_/T_*n*_ mononucleotide repeats are a key hallmark of MMR-deficient cancer cells (2, 3). We have also observed that FAN1 deficiency alone appears to reduce the number of deletions in mononucleotide repeats. However, this was not seen in FAN1-deficient induced pluripotent stem cells (iPSC) or HAP1 cells examined in an independent study (52). This effect is possibly linked to FAN1 interference with DSB metabolism in the TK6 cells, as was seen in the response of the cells to MNU discussed above.

Importantly, the spontaneous mutagenesis increase seen in the *EXO1^−^* and *FAN1*/*EXO1* knockout TK6 cells was substantially lower than that observed in MSH6-deficient HAP1 (52) or iPS cells (56). In the former study, the number of repeat-mediated deletions detected in the parental clone DNA was ∼40, in *EXO1^−^* cells ∼60, and in *MSH6^−^* ∼800. Thus, in agreement with the results of the cell viability assays described above, the lack of both EXO1 and FAN1 does not bring about full MMR deficiency.

## DISCUSSION

The experiments described in this study show that exogenous FAN1 can fully compensate for the absence of EXO1 in an *in vitro* MMR assay and that this function is attenuated when its interaction with MLH1 is abrogated (Fig. 1 and 2). However, this raises the question of why extracts lacking EXO1 are MMR-deficient, when they contain clearly detectable levels of FAN1 (see Fig. S2C in the supplemental material). We considered the possibility that the FAN1/MLH1 complex is required to be posttranslationally modified during S-phase in order to be active. But this is unlikely to be the cause of the lack of FAN1 participation in MMR in the absence of EXO1, given that MLH1 and FAN1 largely comigrate in gel filtration experiments (28), suggesting that the polypeptides are associated in cell extracts, possibly as a heterotrimer FAN1/MLH1/PMS2. An alternative explanation is that FAN1 in complex with MLH1 is inactive in MMR and that FAN1 substitutes for EXO1 only in the free form, i.e., when it is added to the extracts exogenously. But this explanation is also unsatisfactory, given that FAN1 is more active in MMR *in vitro* when associated with MLH1 and that its nuclease activity is not affected by its ability to bind MLH1 (Fig. 2). Interestingly, FAN1 is not the only nuclease that is apparently unable to compensate for EXO1 deficiency. A report by Desai and Gerson (57), using short hairpin RNA (shRNA)-mediated knockdowns, implicated—in addition to FAN1—also Artemis and MRE11 in MMR, as measured by the plasmid-based *in vivo* assay. Clearly, like FAN1, these nucleases do not compensate for EXO1 in this scenario, but, in contrast to FAN1, the evidence in support of their involvement in MMR *in vivo* is questionable. MRE11 functions as part of a heterotrimeric complex with RAD50 and NBS1 in the repair of DSBs, where it is able to endonucleolytically cleave 5′ overhangs. As erroneously stated in that study, it is not a 5′ to 3′ exonuclease; its polarity is 3′ to 5′. Having a polarity opposite to that of EXO1 does not *a priori* preclude MRE11 involvement in MMR, especially when coupled with its ability to digest DNA from a nick (P. Cejka, personal communication). Indeed, bacterial MMR involves several nucleases of both polarities (58), but the mutation of *MRE11* does not bring about microsatellite instability (MSI) as stated (57). The gene is, indeed, frequently mutated in both alleles in cancer, but specifically in MSI-positive tumors that have a driver mutation in a bona fide *MMR* gene (59). This explains why the *MRE11* mutations occur in an intronic T_*n*_ microsatellite. Moreover, the efficiency of the shRNA-mediated knockdown in that study was less than 30%, which was highly unlikely to have had phenotypic consequences such as an increased resistance to the methylating agent temozolomide. The purported link of Artemis to MMR was also unexpected, given that this polypeptide is an endonuclease that has to be activated by ATM and that has not been reported to possess an exonucleolytic activity. Also in this case, the knockdown efficiency was poor (∼50%) and unlikely to have had an effect on the phenotype of the cells. This supposition is based on our demonstration that even ∼90% reduction in the amount of MLH1 was insufficient to render cells MMR-deficient *in vitro* (60).

In our hands, the loss of EXO1 in HAP1 cells did not reduce cell sensitivity to MNU or TG (Fig. 4B and C), and the sensitivity of *EXO1^−/−^* TK6 cells was even increased. This result differs from those obtained with *Exo1^−/−^* MEFs (11) and *Exo1^−/−^ Mgmt^−/−^* MEFs (50), which displayed slight resistance to MNNG. However, the *FAN1^−^* HAP1cells were slightly and the *EXO1^−^*/*FAN1^−^* cells were substantially more resistant to both chemicals than the WT cells, and *FAN1* inactivation rescued the hypersensitivity of the *EXO1^−/−^* cells (Fig. 4A), which can be taken as evidence of the common involvement of the two nucleases in the MMR-dependent processing of the methylated mispairs induced by these chemicals. However, the observed resistance was lower than that seen in the control, MSH6-deficient cells, which suggests that the lack of both nucleases does not bring about full MMR deficiency.

Similarly, the number of mutations in the *FAN1*/*EXO1* double knockout TK6 cells was higher than that in the WT or in the two single mutant lines (Fig. 5) but lower in magnitude than that seen in MSH6-deficient HAP1 (52) or iPS cells (56). Taken together, the above-described evidence suggests that additional antimutagenic mechanisms are at play in protecting eukaryotic genomes from replication errors.

The *in vitro* MMR assay used in this study was first described by the Modrich laboratory using extracts of human and Drosophila melanogaster cells and bacteriophage M13-based heteroduplexes (19). This assay played a key part in the reconstitution of the minimal human MMR system with purified proteins (21, 22, 61). During the course of those studies, EXO1 was shown to play a key role in the reconstituted system (15). Importantly, in subsequent experiments using extracts of *Mlh1^−/−^ Exo1^−/−^* mouse embryonic fibroblasts (MEFs), Kadyrov and colleagues (38) restored efficient MMR by the addition of recombinant MutLα and EXO1 but detected low levels of nick-dependent mismatch correction also in extracts supplemented with MutLα alone. Because the latter repair was inhibited by the addition of aphidicolin, they postulated that assembly of the EXO1-deficient MMR repairosome on the heteroduplex activated the strand displacement activity of polymerase δ, which displaced the error-containing fragment of the nicked DNA strand to generate a single-stranded flap that was subsequently cleaved off by FEN1.

The alternative mechanism discussed above does not function efficiently in cell extracts; when the same heteroduplex was incubated with the reconstituted system lacking EXO1, repair efficiency was substantially greater than that seen in the complemented *Mlh1^−/−^ Exo1^−/−^* MEF extracts (38). However, it may be substantially more efficient in an *in vivo* setting and could obviate the requirement for additional exonucleases in MMR. This will be challenging to test experimentally, given that no variants of the polymerase δ protein complex lacking strand displacement activity have been characterized, at least not to our knowledge. But the finding that *exo1* mutations in S. cerevisiae are synthetically lethal in combination with those in the *rad27* locus that encodes FEN1 (9) may provide indirect evidence in support of the strand displacement mechanism working in tandem with EXO1-dependent MMR.

In summary, the evidence presented in this study implicates FAN1 as a nuclease that can compensate—at least partially—for the lack of EXO1 in MMR. However, given that the loss of FAN1 alone significantly affects spontaneous mutagenesis neither in human cells (this study) nor in Schizosaccharomyces pombe (62), while it gives rise to hypersensitivity to ICL-inducing agents (24, 26, 28, 29), we conclude that the primary biological role of FAN1 lies in the processing of secondary structures such as ICLs, rather than in MMR. As its name implies, FAN1 interacts with components of the Fanconi anemia group of proteins and there is little doubt that it participates in this pathway. That the *FAN1* gene has not been found to be mutated in Fanconi anemia (FA) patients is most likely due to its functional redundancy with other nucleases in the pathway, such as SLX1 and/or SNM1A, neither of which is, like FAN1, encoded by an *FA* gene (see reference 25 for a recent review). Based on our findings, it appears highly probable that, as in the case of *EXO1*, mutations in the *FAN1* gene will also not segregate with cancer. However, FAN1 appears to play an antimutagenic role in triplet repeat expansion disease (TRD); the *FAN1* locus on chromosome 15 was clearly identified by a genome-wide association study (GWAS) as a modifier of Huntington’s disease (HD). Inactivating mutations were linked to precocious onset of disease, whereas overexpression was protective (63). The *FAN1* locus has also been linked to a second TRD, fragile X syndrome (64). Interestingly, inactivating *MMR* gene polymorphisms—primarily in *MSH3* and *MLH1*—have also been linked to TRD, mainly as accelerators of HD onset (63, 65). This suggests that the MMR repairosome recognizes (likely via the MSH2/MSH3 heterodimer) and aberrantly incises a subset of secondary structure motifs arising during DNA metabolism at the repeat motifs, which leads to their expansion. In the absence of FAN1, these incisions become more frequent, possibly because FAN1 (particularly when overexpressed) may limit the action of the other nucleases associated with MLH1, namely, PMS2, MLH3, and EXO1. This hypothesis is supported by the most recent findings that the expansion of CAG repeats in *Fan1^−/−^* HD knock-in mice was blocked by disruption of *Mlh1* (66). Thus, in the TRD scenario, FAN1 appears to have the opposite function to that in canonical MMR, where it can act as a backup for EXO1.

## MATERIALS AND METHODS

### Reagents and methods.

*DT-ApA*/*MARKER^R^* was provided by the Laboratory for Animal Resources and Genetic Engineering, Center for Developmental Biology, RIKEN Kobe (http://www2.clst.riken.jp/arg/Cassette/CassetteMap_16.html). The antibodies used were anti-MLH1 antibody (ab92312; Abcam; 1:1,000 in 1% milk–Tris-buffered saline with Tween 20 [TBST]), anti-β-tubulin (ab6046; Abcam; 1:1,000 in 5% milk–TBST), anti-EXO1 antibody (Bethyl; A302-639; 1/1,000 in 1% milk–TBST), anti-PCNA (Santa Cruz; sc-56; 1:1,000 in 1% milk–TBST), anti-rabbit IgG horseradish peroxidase (HRP)-linked whole antibody (GE Healthcare UK Limited; 1:10,000 in 5% milk–TBST), goat anti-FAN1 antibody (1:100 in 1% milk–TBST; a kind gift from John Rouse, University of Dundee), anti-goat IgG HRP-conjugated antibody (1:5,000 in 5% milk–TBST).

### Cell lines.

Chicken B-lymphocyte DT40 cells were cultured in RPMI 1640 medium containing 2 mM l-glutamine, supplemented with 10% heat-inactivated fetal bovine serum (FBS), 1% chicken serum (all from GIBCO-BRL), and 50 μM β-mercaptoethanol at 39.5°C in 5% CO_2_ were used. Human embryonic kidney (HEK) 293 cells were cultured in Dulbecco’s modified Eagle’s medium (DMEM) containing 2 mM l-glutamine (GIBCO-BRL) supplemented with 10% FBS, 10,000 U/ml penicillin, and 10 mg/ml streptomycin (GIBCO-BRL) was used. HEK 293TLα(+/−) cells were cultured as previously described (60). To inhibit expression of MLH1, doxycycline (50 ng/ml; Abcam) was added to HEK 293TLα(−) cells. The human TK6-derived lymphoblastoid cell line TSCER2 (67) cells were grown in an RPMI 1640 medium supplemented with 5% heat‐inactivated horse serum or 10% FBS, 200 μg/ml sodium pyruvate, 100 U/ml penicillin, and 100 μg/ml streptomycin. Human HAP1 cells were grown in Iscove’s modified Dulbecco’s medium (IMDM; GIBCO-BRL), containing l-glutamine and 25 mM HEPES, and supplemented with 10% FBS, 10,000 U/ml penicillin, and 10 mg/ml streptomycin. The cells were diploid at the time of the experiments. All human cell lines were cultured at 37°C in 5% CO_2_.

### Construction of DT40 gene targeting vectors.

The region of the *G. gallus MLH1* gene containing exon 6 was PCR-amplified using the primer pairs listed in Table S1 in the supplemental material. An additional SphI restriction site and a stop codon were introduced at the end of the left vector arm. The amplified 3.6-kb left- and 4.1-kb right arms were sequentially subcloned into pBlueScript SK+ (Stratagene) using the restriction sites XhoI-BamHI and BamHI-NotI, respectively. The *puro* and *bsr* selection marker genes flanked by loxP sequences and the *hisD* selection marker gene without flanking loxP sequences were inserted into the BamHI restriction site of the vector to generate MLH1-puro, MLH1-bsr, and MLH1-hisD targeting constructs. The constructs were linearized with NotI prior to transfection into chicken B-lymphocyte DT40 cells.

### Generation of MLH1-deficient DT40 cells.

The cells were sequentially transfected by electroporation (Bio-Rad) with *MLH1*-*puro*, *MLH1-bsr*, and *MLH1-hisD* targeting constructs (Fig. S1A) to obtain *MLH1*^−/−/−^ cells. Gene targeting events were verified by the appearance of 6.5-, 8.9-, and 8.3-kb DNA fragments and the disappearance of a 12.2-kb DNA WT fragment in Southern blot analysis of SphI-digested genomic DNA (Fig. S1B). A 1.1-kb DNA fragment generated by PCR amplification of the *MLH1* gene was used as the probe. Gene disruption was confirmed by RT-PCR analysis. β-Actin was used as the positive control for RT-PCR (Fig. S1C). See Table S1 for all primer sequences.

### Generation of FAN1/EXO1-deficient DT40 cells.

The *puro* and *bsr* resistance genes in the DT40 *FAN1^−/−^* cells (24) were first “floxed out” with *Cre* recombinase. Antibiotic-sensitive clones were then sequentially transfected by electroporation with *EXO1*-*puro* and *EXO1-bsr* targeting constructs (Fig. S1D) (34) to obtain *FAN1^−/−^ EXO1*^−/−^ cells. Following selection with the appropriate antibiotics, gene targeting events were verified by the appearance of two 6.5-kb DNA fragments and the disappearance of a 10.5-kb DNA WT fragment in Southern blot analysis of ApaLI and BamHI digested genomic DNA (Fig. 1E). A 350-bp DNA fragment generated by PCR amplification of the *EXO1* gene was used as the probe. Gene disruption was confirmed by RT-PCR, analysis and β-actin transcripts were analyzed as a positive control (Fig. 1F). See Table S1 for all primer sequences.

### Cell extracts.

The nuclear extracts of human cells were prepared as described previously (36). The DT40 extracts were generated as follows: 200 × 10^6^ cells were harvested (5 min at 1,000 rpm and 4°C), washed once in phosphate-buffered saline (PBS), and resuspended in 350 μl lysis-buffer (10 mM Tris-HCl [pH 7.5], 2 mM MgCl_2_, 3 mM CaCl_2_, 0.32 M sucrose, 1 mM dithiothreitol [DTT], 0.1 mM spermine, 0.5 mM spermidine, 1× complete EDTA-free protease inhibitor cocktail [Roche]). After addition of 0.3% IGEPAL-CA630 (Sigma), the suspension was gently mixed and immediately centrifuged for 10 min at 20,000 × *g* and 4°C. The supernatant (total cell extract) was aliquoted, snap-frozen in liquid nitrogen, and stored at −80°C.

### Generation of FAN1-, EXO1-, and MLH1-deficient TK6 and HAP1 cells.

Guide RNA (gRNA) target sequences were designed using an online tool provided by the Zhang Feng laboratory (68). The complementary gDNA oligonucleotides for EXO1 and MLH1 (Table S1) were annealed and cloned into a BbsI site of pX330, which expresses gRNA and Cas9 from the U6 and chicken β-actin promoters, respectively. To generate the TALEN expression plasmids for the *FAN1* gene, a Golden Gate TAKEN kit and a TAL effector kit (Addgene) were used. The effector module was set for 5′-TAGAAGCTTATCAATCAG-3′ and 5′-AGCATCTAATTCTATTA-3′ in exon 1. To generate the targeting vectors, left and right homology arms (∼1 kb) were amplified by PCR using TK6 genomic DNA as the template with forward right/reverse right and forward left/reverse left primer pairs, respectively (Table S1). The *FAN1* targeting vector (Fig. S2D) was assembled with *DT-ApA*/*PURO^R^* and *DT-ApA*/*HYGRO^R^* vectors using GeneArt seamless cloning enzyme mix (Thermo Fischer, USA) as described previously (34). *T*he *EXO1* targeting vector (Fig. S2F) was assembled with *DT-ApA*/*NEO^R^* and *DT-ApA*/*PURO^R^* vectors were assembled in the same manner. The *FAN1* and *EXO1* knockout cell lines were generated by electroporating 6 × 10^6^ TK6 cells (Neon system; Invitrogen; 1,350 V, 10 ms, 3 pulses, 100-ml Neon tip) with 2 μg of the appropriate linearized targeting vectors, together with 6 μg of the respective pX330 Cas9/gRNA-expressing plasmids. After 48 h of incubation in fresh medium, the transfectants were incubated for 2 weeks with the appropriate antibiotics in 96-well plates (puromycin, 0.5 mg/ml; G418, 1 mg/ml; and hygromycin B, 0.625 mg/ml dissolved in 10 mM HEPES, pH 7.5). The resistant clones were characterized by *S*outhern blotting. The probes were generated using the primers shown in Table S1. Genomic DNA was digested with HindIII (Fig. S2E and G). Expression of FAN1, EXO1, and MLH1 was analyzed by Western blotting (Fig. S2H).

The EXO1-deficient HAP1 cell line was described earlier (69). The *FAN1^−^* and *FAN1^−^ EXO1^−^* double knockout cell lines were generated analogously to the respective TK6 knockout (KO) cell lines. The gene disruptions were verified as described above and by whole-genome sequencing.

### Expression and purification of recombinant human and chicken proteins.

The recombinant human EXO1 and the nuclease-dead mutant EXO1 D173A were obtained as described previously (35). The human FAN1 and nuclease-dead mutant FAN1 D960A were described elsewhere (26). The L155A,L159A and the gFAN1 DA variants were obtained using an identical procedure. The 3.2-kb chicken FAN1 cDNA was amplified from a DT40 cDNA library using the primer pair 5′-GCC GCG GAA AGG CTT TGA AGT TCC-3′ and 5′-CTG GTA GCG TGT AGC ATG TCC C-3′ and cloned into pFastBak containing an N-terminal MBP tag, which was cleaved off with TEV protease according to the manufacturer’s instructions (New England Biolabs [NEB]). Site-directed mutagenesis was carried out using the QuikChange kit (Agilent).

### Generation and characterization of HEK293 EXO1 knockdown cells.

EXO1 knockdown was carried out as described previously (35) using siRNA 5′-CAAGCCUAUUCUCGUAUUUTT-3′ (Microsynth). The transfected cells were harvested after 72 h and the efficiency of EXO1 knockdown was determined by Western blotting (see also Fig. S2C).

### Mismatch repair substrates.

The heteroduplex DNA substrate for the *in vitro* MMR assays was generated by primer extension, using the oligonucleotide 5′-GGCCGCGATCTGATCAGATCCAGACGTCTGTCGACGTTGGGAAGCTTGAG-3′ and a single-stranded phagemid pGEM13Zf(+)DNA template as previously described (36). The substrate for the *in vivo* MMR assay was generated by annealing pmCherry plasmid (Clonetech) linearized with BsaI-HF (NEB) with TA pmCherry single-stranded (ssDNA) as described previously (41) A detailed protocol is described in supplemental material. The substrate was purified by CsCl gradient ultracentrifugation at 60,000 rpm for 16 h (Sorvall 65V13 rotor) at room temperature.

### *In vitro* MMR assay.

Assays were carried out as described previously (36). Briefly, the nicked heteroduplex phagemid DNA substrate (48 fmol) (Fig. 1A) was incubated with 100 μg of cell extracts in 20 mM Tris-HCl [pH 7.6], 110 mM KCl, 5 mM MgCl_2_, 1 mM glutathione, 1.5 mM ATP, 50 μg/ml BSA, and 100 μM dNTPs for 30 min in a total volume of 25 μl. The reactions were terminated by a 30-min incubation with a stop solution (final concentration of 0.5 mM EDTA, 1.5% SDS, 2.5 mg/ml proteinase K) and cleaned up on a MinElute column (Qiagen), and the recovered phagemid was digested with AclI (NEB). RNase A (Sigma-Aldrich) was then added and, following an overnight incubation at 37°C, the reaction products were separated on a 1% agarose gel eluted with Tris-acetate-EDTA (TAE) buffer and stained with GelRed.

### *In vivo* MMR assay.

The nicked T/G pmCherry plasmid and pEGFP (as a transfection control) were cotransfected into TK6 cell lines (WT, *FAN1^−/−^*, *EXO1^−/−^*, and *FAN1^−/−^ EXO1^−/−^*) using the Nucleofector transfection machine and kit (Amaxa). The *MLH1^−/−^* TSCER-2 cell line was used as an MMR-deficient control. For each cell line variant, 5 × 10^6^ cells were transfected with 2 μg of each plasmid using program A30. After 24 h, a minimum of 5,000 cells were measured for their fluorescence intensity at 530 nm (EGFP) and 610 nm (mCherry) using an LSRFortessa flow cytometer (Becton, Dickinson). Data were plotted using FlowJo software, and the ratio between mCherry- and EGFP-expressing cells was defined as the percentage of repair.

### Clonogenic assays.

MNU sensitivity of TK6 suspension cells was tested in methylcellulose (Sigma-Aldrich) semisolid medium (70). Briefly, sterilized methylcellulose suspended in warm water (3% wt/vol) was mixed with an equal volume of 2× DMEM–F-12 medium (1:1; Gibco). After overnight stirring, FBS (10% final concentration) and sodium pyruvate (200 μg/ml final concentration) were added. Freshly prepared solution of MNU in DMSO was serially diluted in the methylcellulose-medium mix and rotated at 4°C overnight. Then, 300 to 1,500 cells/well were seeded in 6-well plates and mixed with 3 ml of the methylcellulose-medium mix, and the MNU-methylcellulose-medium mix was added to yield the final MNU concentrations shown in Fig. 4A. Following thorough mixing, the plates were allowed to incubate at 37°C for 10 to 14 days, and the colonies were counted. Survival was calculated relative to the untreated controls.

### Cell survival assays.

Six thioguanine survival assays were performed in three independent experiments by plating 0.5 × 10^3^ HAP1 cells per well of flat-bottom 96-well plates in triplicate for each condition. In the case of MNU survival assays, the cells were pretreated with 10 μM 6-benzylguanine (Sigma-Aldrich) for 2 h. The indicated amounts of TG (Sigma-Aldrich) and MNU (MedChem Express) were added to the wells on the day of plating prior to cell seeding. Cell viability was measured 7 days after plating using Cell-Titer Glo (Promega) following the manufacturer’s instructions (1:4 dilution in water and incubation for 20 min. at room temperature). Luminescence was measured with an Infinite 200 Pro plate reader (Tecan).

### Mutational signature analysis.

The TSCER-2 WT, *FAN1^−/−^*, *EXO1^−/−^*, and *FAN1^−/−^ EXO1^−/−^* cell lines were grown under optimal growth conditions (see above) to estimate their doubling times, which were 13, 15, 14, and 16 h, respectively. Aliquots of these cell populations (population 0 [p0]) were isolated, and their DNA was extracted for sequencing. Ten single cells from each p0 population were then grown for exactly 40 generations. The DNA from three clones of each p40 generation was extracted for sequencing. The sequencing analysis was carried out as described previously (52).

### Data availability.

The genome sequences have been deposited in GenBank for Homo sapiens NP_000240.1 (MLH1), *Gallus repair some* XP_418828.1 (MLH1), H. sapiens CAI15658.1 (EXO1), *G. gallus* XP_419550.3 (EXO1), H. sapiens NP_055782.3 (FAN1), and *G. gallus* XP_413768.2 (FAN1).
