# Supplementary material for: FANCD2-Associated Nuclease 1 Partially Compensates for the Lack of Exonuclease 1 in Mismatch Repair
Source: Mol Cell Biol. 2021 Aug 24;41(9):e00303-21. doi: 10.1128/MCB.00303-21 (PMC8384067; doi:10.1128/MCB.00303-21)
Supplement: Supplemental file 1 — Supplemental text, Table S1, and Fig. S1 to S3. Download MCB.00303-21-s0001.pdf, PDF file, 2.3 MB [file mcb.00303-21-s0001.pdf]

Supplementary Information

Supplementary Materials and Methods

Mismatch repair substrates – The substrate for the *in vivo* assay (1) was generated from the pmCherry-C1 plasmid (Clontech). First, a Nb.*Bsr*DI nicking site was generated by PCR mutagenesis, using the primers $^{5'}$GTG GGA GGT CTA TAT AAG CAA TGC TGG TTT AGT GAA CCG TCA$^{3'}$ and $^{5'}$CTG ACG GTT CAC TAA ACC AGC ATT GCT TAT ATA GAC CTC CCA C$^{3'}$. The resulting plasmid was named CG pmCherry. PCR mutagenesis of CG pmCherry and the primers $^{5'}$CTT CCC CGA GGG CTT CAA GTA GGA GCG CGT GAT GAA CTT CG$^{3'}$ and $^{5'}$CGA AGT TCA TCA CGC GCT CCT ACT TGA AGC CCT CGG GGA AG$^{3'}$ was then used to generate a mutant, TA pmCherry, which contained a premature stop codon (TGG_{98}--> TAG) in the mCherry ORF that completely eliminated the fluorescent properties of mCherry (data not shown). Since pmCherry vectors contain an f1 origin of replication, circular ssDNA (the viral, T strand) could be obtained from the TA pmCherry plasmid as described (2).

Next, CG pmCherry was linearized with *Bsa*I-HF (NEB) and cleaned-up with MiniElute Clean up kit (QIAGEN). Aliquots of 4.5 µg of the linear DNA were diluted with MilliQ water to 50 µl final volume and heated at 85ºC for 15 min. TA pmCherry ssDNA was then added at a ratio 1:3 and the mixture was heated for 2 min more. After incubation for 5 min on ice, 20 x SSC buffer was added to 2x final concentration (300 mM NaCl, 30 mM Na Citrate, pH 7) and the sample was heated at 60ºC for 10 min. Efficiency of the annealing reaction was checked on 1% agarose gels stained with Gel-Red. Afterwards, 20 µg of the mixture was incubated with 50 U of Nb.*Bsr*DI (NEB) for 1.5 h at 65ºC to introduce a nick in the T strand 340 nucleotides 3' from the mismatch. The sample was then digested with Plasmid Safe ATP-dependent DNase (Epicentre Biotechnologies) to degrade the excess linearized dsDNA. The nicked T/G pmCherry plasmid substrate was cleaned up with MiniElute Clean up kit and stored at -20˚C.

**Supplementary Table S1**
Oligonucleotides for the construction of DT40 targeting vectors
MLH1, Forward primer left arm, 5'-GTTGCCCATGTTACGGTAACAACTAAAACAGC-3'
MLH1, Reverse primer left arm, 5'-AGCTTTCCTCCTTGTATTTACATTGTAAAAAAGATCTTCC
MLH1, Forward primer right arm, 5'-CGAGTGAAGAGTATGCAAAAATACTAGAAGTTG-3'
MLH1, Reverse primer right arm, 5'-GAAGTACATCCTTGAAGAATTGGAACCC-3'
Oligonucleotide probes for DT40 Southern blotting
MLH1, Forward, 5'-CCAGTGAGGTGGTAAAATCAGCAGCAAG-3'
MLH1, Reverse, 5'-CTTCCATTTGTATGTCTGTGTCTTCCCG-3'
Oligonucleotides for DT40 RT-PCR
MLH1, Forward, 5'-GGTTTTAGGGGTGAGGCATTGG-3'
MLH1, Reverse, 5'-GGGGGGGCTTTGATTTTTCC -3'

b-actin, Forward, 5’-GTGCTGTGTTCCCATCTATCGTG-3’
[illegible]-actin, Reverse, 5’-GACAATACCGTGTTCAATGGGG-3’
TK6 gRNA sequences
FAN1, 5’- CACCGCTGATTGATAAGCTTCTACG-3’
FAN1, 5’-AAACCGTAGAAGCTTATCAATCAGC-3’
EXO1, 5’- CACCGTCAGGGGGTAGATTGCCTCG-3’
EXO1, 5’-AAACCGAGGCAATCTACCCCCTGAC-3’
Oligonucleotides for the construction of TK6 targeting vectors
FAN1, Forward primer arm, 5’- GCGAATTGGGTACCGGGCCAAAACCCAGCTCAGTGGTTTTCCAACTGAA -3’
FAN1, Reverse primer arm, 5’- CTGGGCTCGAGGGGGGGCCTCATGAGTATTAGAAAAACTGGATGTTCTGAGCAAT -3’
FAN1, Forward primer right arm, 5’- TGGGAAGCTTGTCGACTTAATTTCGTGTTTTAACAATGCACCACCTGCTA -3’
FAN1, Reverse primer right arm, 5’- CACTAGTAGGCGCGCCTTAAAAAGAGCAACATATCATCTTCATTCTCAAGTACGG -3’
EXO1, Forward primer left arm, 5’-GCGAATTGGGTACCGGGCCCTTCTCAAAAGGATGAGAGT-3’
EXO1, Reverse primer left arm, 5’-CTGGGCTCGAGGGGGGGCCAAGAGAGAATCACAGTGAAT-3’
EXO1, Forward primer right arm, 5’-TGGGAAGCTTGTCGACTTAAGTACTCACCTCTGACTACTA-3’
EXO1, Reverse primer right arm, 5’-CACTAGTAGGCGCGCCTTAACTATATCTGGATTATTGGCT-3’
Oligonucleotide probe for TK6 Southern blotting
FAN1, Forward, 5’- ACATTGGAAGCAGCTGTTTTTCTTTTATAG-3’
FAN1, Reverse, 5’- CCAACCAAAGATCTGTGACTAGTTTGGATA-3’
EXO1, Forward, 5’-CTCTCACAGAAATCAGAATGTCAACTTGGT-3’
EXO1, Reverse, 5’-TGCCCAGCGTAGCTTAGTGTTTCAGGATCA-3’
Oligonucleotides for RT-PCR
FAN1, Forward, 5’-ATG ATG TCA GAA GGG AAA CCT CCT GAC AAA-3’
FAN1, Reverse, 5’-AGC TTG TCG ACT TAA TTA GCT AAG GCT TTG GCT CTT AGC TCC AAC-3’
EXO1, Forward, 5’-ACAAAGTAATTAAAGCTGCC-3’
EXO1, Reverse, 5’-CTGACAGCTCTGCACTTCTTGGTC-3’
PCNA, Forward, 5’-ATGTTCGAGGCGCGCCTGGT-3’
PCNA, Reverse, 5’-CTAAGATCCTTCTTCATCCT-3’

Supplementary Figure Legends

Supplementary Figure S1. (**A**) Schematic representation of the chicken *MLH1* gene and the fragment sizes resulting from *Sph*I digestion of genomic DNA following a correct insertion of the targeting vectors containing the three antibiotic resistance genes. (**B**) Southern blot analysis of genomic DNA of WT DT40 cells and the clones in which one, two or three alleles were disrupted. (**C**) Western blot of extracts of the clones shown in B. (**D**) Schematic representation of the chicken *EXO1* gene and the fragment sizes resulting from *Bam*HI digestion of genomic DNA following a correct insertion of the targeting vectors containing the two antibiotic resistance genes. (**E**) Southern blot analysis of genomic DNA of WT DT40 cells and the clones in which one, two or three alleles were disrupted. (**F**) Western blot of extracts of the clones shown in E. (**G**) Coomassie blue-stained SDS-PAGE (10%) of nuclear extracts of human HEK293T-Lα (Lα+) and TK6 (B cells), as well DT40 cells.

NEa, nuclear extract made by standard protocol; CE, cytosolic extract; NE, nuclear extracts made by the modified protocol. (**H**) Agarose gel (0.8%) electrophoresis of the samples in G. The gel was stained with GelRed.

Supplementary Figure S2. (**A**) Western blot of a GFP pull-down assay. Extracts of HEK 293 cells expressing either GFP-FAN1 or its LALA variant were incubated with GFP-Trap beads as described previously (3). The eluted proteins were separated by SDS-PAGE (10%), transferred to a membrane and blotted with the indicated antibodies. The blot shows that the interaction of FAN1-LALA variant with endogenous MLH1 is much weaker that with that of the WT FAN1 polypeptide. (**B**) Coomassie blue-stained SDS-PAGE (10%) of purified recombinant human FAN1 and its LALA and DA variants. (**C**) Western blot of extracts of HEK293 treated with siRNA against luciferase (siLuc) or EXO1 (siEXO1). The blots were stained with the indicated antibodies. (**D**) Schematic representation of the human *FAN1* gene and the fragment sizes resulting from *Hin*dIII digestion of genomic DNA following a correct insertion of the targeting vectors containing the antibiotic resistance gene. (**E**) Southern blot analysis of genomic DNA of WT and *FAN1*$^{-/-}$ TK6 cells. (**F**) Schematic representation of the human *EXO1* gene and the fragment sizes resulting from *Hin*dIII digestion of genomic DNA following a correct insertion of the targeting vectors containing the antibiotic resistance gene. (**G**) Southern blot analysis of genomic DNA of WT and *EXO1*$^{-/-}$ TK6 cells. (**H**) Western blot analysis of extracts of WT, *FAN1*$^{-/-}$, *EXO1*$^{-/-}$ and *FAN1*$^{-/-}$ *EXO1*$^{-/-}$ TK6 cells.

Supplementary Figure S3. (**A**) Western blot analysis of extracts of WT, *EXO1*$^{-/-}$ and two clones obtained upon transfection of *EXO1*$^{-/-}$ cells with an pIRES-EGFP-EXO1 expression vector. (**B**) Survival assay indicating that the expression of exogenous EXO1 in *EXO1*$^{-/-}$ clones 2 and 22 (see panel A) rescued their hypersensitivity to MNU. (**C**) FAN1 cleaves open-circular DNA opposite the pre-existing nick. The plasmid substrate and the fragments generated upon its incubation with increasing amount of recombinant FAN1 are indicated in the scheme below the 0.8% agarose gel stained with GelRed. M, 1 kb marker (BioRad). (**D**) Survival assay showing the sensitivity of WT, *FAN1*$^{-/-}$, *EXO1*$^{-/-}$ and *FAN1*$^{-/-}$ *EXO1*$^{-/-}$ TK6 cells to the ICL-inducing agent MMC.

References

1. Zhou, B., Huang, C., Yang, J., Lu, J., Dong, Q. and Sun, L.Z. (2009) Preparation of heteroduplex enhanced green fluorescent protein plasmid for in vivo mismatch repair activity assay. *Anal Biochem*, **388**, 167-169.
2. Baerenfaller, K., Fischer, F. and Jiricny, J. (2006) Characterization of the "mismatch repairosome" and its role in the processing of modified nucleosides in vitro. *Methods Enzymol*, **408**, 285-303.
3. Porro, A., Berti, M., Pizzolato, J., Bologna, S., Kaden, S., Saxer, A., Ma, Y., Nagasawa, K., Sartori, A.A. and Jiricny, J. (2017) FAN1 interaction with ubiquitylated PCNA alleviates replication stress and preserves genomic integrity independently of BRCA2. *Nat Commun*, **8**, 1073.

Figure S1

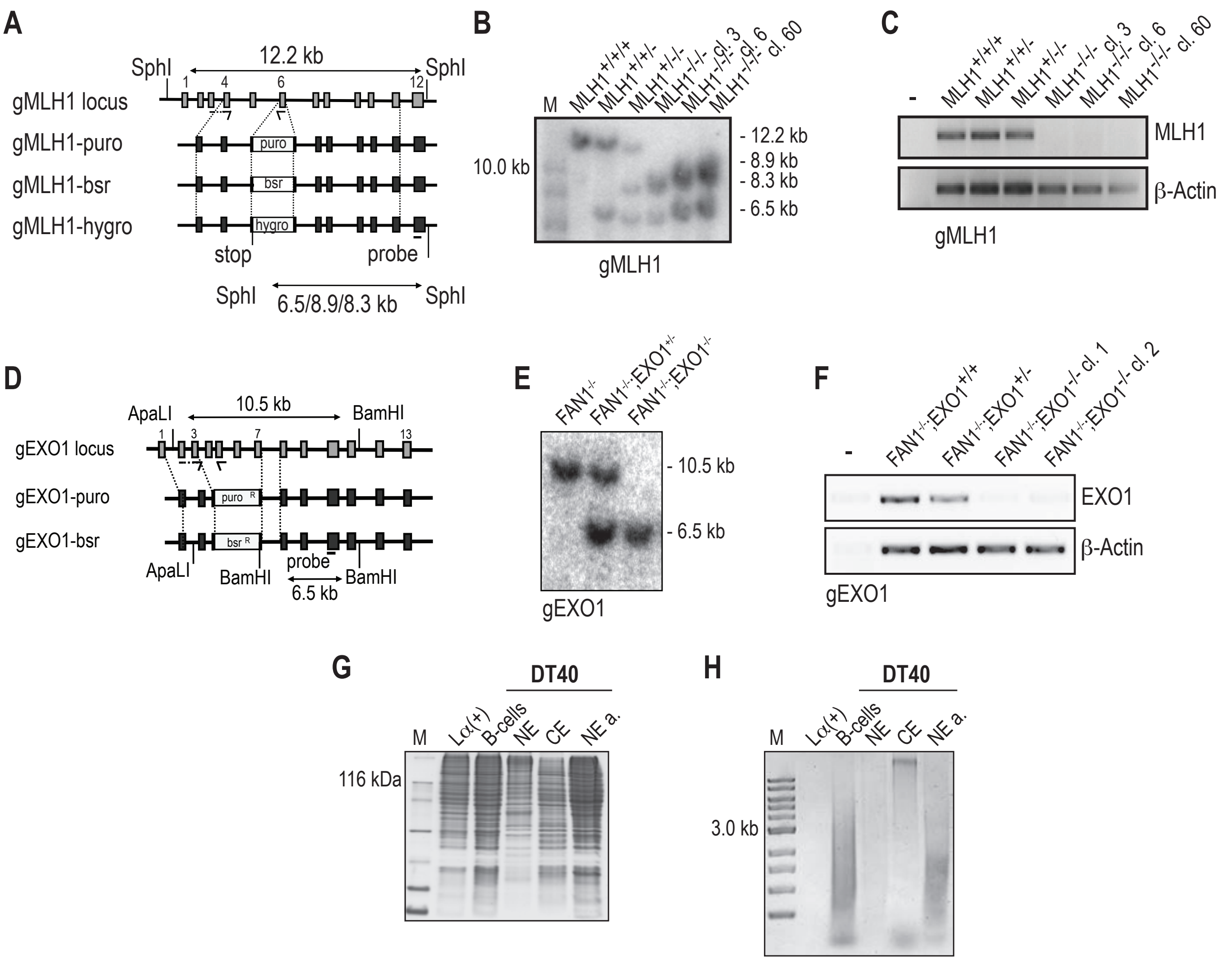

A
SphI
12.2 kb
SphI
gMLH1 locus
gMLH1-puro
gMLH1-bsr
gMLH1-hygro
puro
bsr
hygro
stop
probe
SphI
6.5/8.9/8.3 kb
SphI
B
M
10.0 kb
- 12.2 kb
- 8.9 kb
- 8.3 kb
- 6.5 kb
gMLH1
C
MLH1
β-Actin
gMLH1
D
ApaLI
10.5 kb
BamHI
gEXO1 locus
gEXO1-puro
gEXO1-bsr
ApaLI
BamHI
probe
6.5 kb
BamHI
E
- 10.5 kb
- 6.5 kb
gEXO1
F
EXO1
β-Actin
gEXO1
G
DT40
M
Lα(+)
B-cells
NE
CE
NE a.
116 kDa
H
DT40
M
Lα(+)
B-cells
NE
CE
NE a.
3.0 kb

Fig. S2

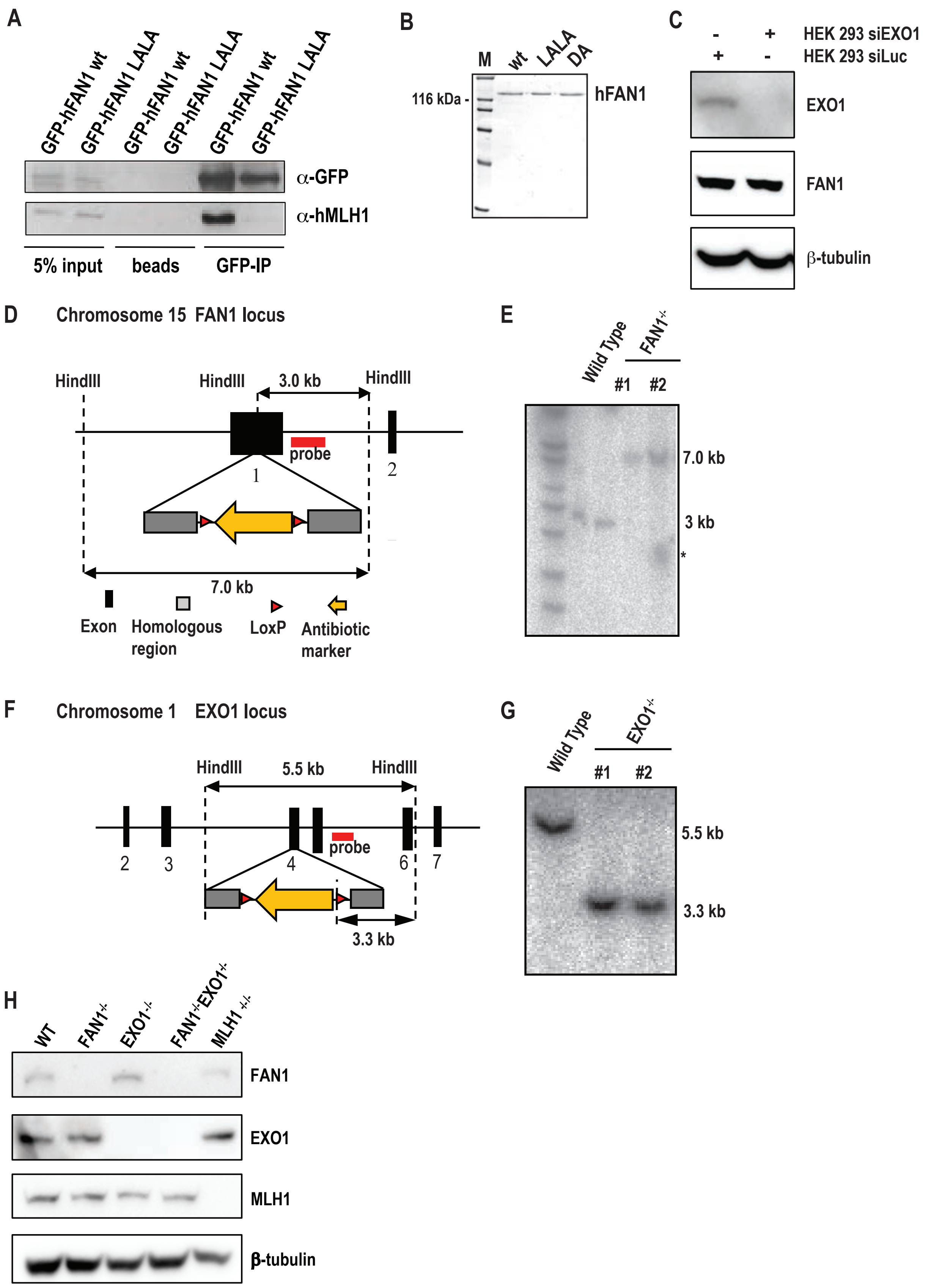
A
GFP-hFAN1 wt
GFP-hFAN1 LALA
GFP-hFAN1 wt
GFP-hFAN1 LALA
GFP-hFAN1 wt
GFP-hFAN1 LALA
α-GFP
α-hMLH1
5% input
beads
GFP-IP
B
M
wt
LALA
DA
116 kDa -
hFAN1
C
- +
HEK 293 siEXO1
+ -
HEK 293 siLuc
EXO1
FAN1
β-tubulin
D
Chromosome 15 FAN1 locus
HindIII
HindIII
3.0 kb
HindIII
probe
1
2
7.0 kb
Exon
Homologous region
LoxP
Antibiotic marker
E
Wild Type
FAN1-/-
#1
#2
7.0 kb
3 kb
*
F
Chromosome 1 EXO1 locus
HindIII
5.5 kb
HindIII
probe
2
3
4
6
7
3.3 kb
G
Wild Type
EXO1-/-
#1
#2
5.5 kb
3.3 kb
H
WT
FAN1-/-
EXO1-/-
FAN1-/-EXO1-/-
MLH1-/-
FAN1
EXO1
MLH1
β-tubulin

Fig. S4

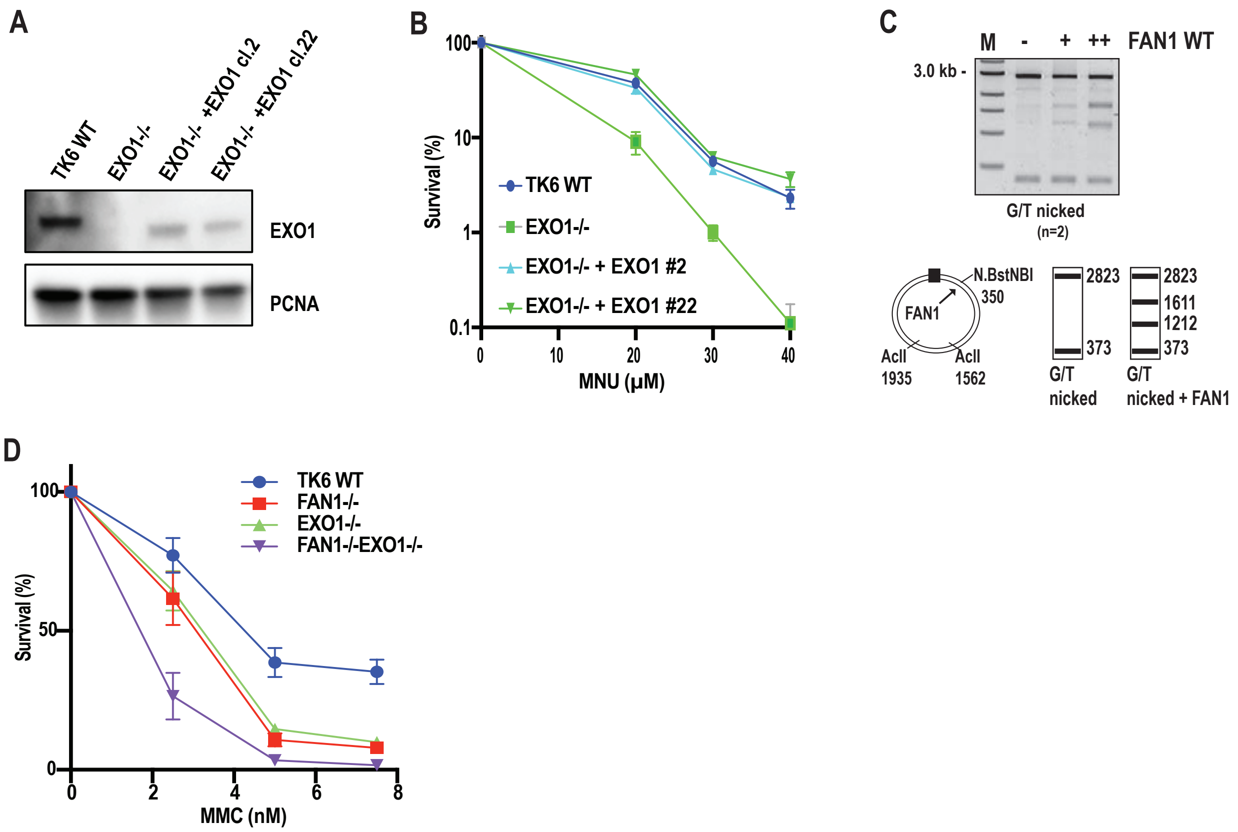
A
TK6 WT
EXO1-/-
EXO1-/- +EXO1 cl.2
EXO1-/- +EXO1 cl.22
EXO1
PCNA
B
Survival (%)
100
10
1
0.1
TK6 WT
EXO1-/-
EXO1-/- + EXO1 #2
EXO1-/- + EXO1 #22
0
10
20
30
40
MNU (μM)
C
M
-
+
++
FAN1 WT
3.0 kb -
G/T nicked
(n=2)
N.BstNBI
350
FAN1
AclI
1935
AclI
1562
2823
373
G/T
nicked
2823
1611
1212
373
G/T
nicked + FAN1
D
Survival (%)
100
50
0
TK6 WT
FAN1-/-
EXO1-/-
FAN1-/-EXO1-/-
0
2
4
6
8
MMC (nM)
